# Role of Spectraplakin in *Drosophila* Photoreceptor Morphogenesis

**DOI:** 10.1371/journal.pone.0025965

**Published:** 2011-10-12

**Authors:** Uyen Ngoc Mui, Christina M. Lubczyk, Sang-Chul Nam

**Affiliations:** Department of Biology, Baylor University, Waco, Texas, United States of America; University of Dayton, United States of America

## Abstract

**Background:**

Crumbs (Crb), a cell polarity gene, has been shown to provide a positional cue for the apical membrane domain and adherens junction during *Drosophila* photoreceptor morphogenesis. It has recently been found that stable microtubules in developing *Drosophila* photoreceptors were linked to Crb localization. Coordinated interactions between microtubule and actin cytoskeletons are involved in many polarized cellular processes. Since Spectraplakin is able to bind both microtubule and actin cytoskeletons, the role of Spectraplakin was analyzed in the regulations of apical Crb domain in developing *Drosophila* photoreceptors.

**Methodology/Principal Findings:**

The localization pattern of Spectraplakin in developing pupal photoreceptors showed a unique intracellular distribution. Spectraplakin localized at rhabdomere terminal web which is at the basal side of the apical Crb or rhabdomere, and in between the adherens junctions. The *spectraplakin* mutant photoreceptors showed dramatic mislocalizations of Crb, adherens junctions, and the stable microtubules. This role of Spectraplakin in Crb and adherens junction regulation was further supported by *spectraplakin*'s gain-of-function phenotype. Spectraplakin overexpression in photoreceptors caused a cell polarity defect including dramatic mislocalization of Crb, adherens junctions and the stable microtubules in the developing photoreceptors. Furthermore, a strong genetic interaction between *spectraplakin* and *crb* was found using a genetic modifier test.

**Conclusions/Significance:**

In summary, we found a unique localization of Spectraplakin in photoreceptors, and identified the role of *spectraplakin* in the regulation of the apical Crb domain and adherens junctions through genetic mutational analysis. Our data suggest that Spectraplakin, an actin-microtubule cross-linker, is essential in the apical and adherens junction controls during the photoreceptors morphogenesis.

## Introduction

*Drosophila* has been extensively used to study the localization and function of cell polarity proteins [1]. Genetic analysis has identified three groups of cell polarity genes that are required for the organization of apical basal epithelial cell polarity. The formation of cell polarity is dependent on the Crumbs (Crb) complex of Crb, Stardust and Patj, the Par complex of Bazooka (Baz, Par-3), Par-6 and a typical protein kinase C (aPKC), and the Scrib complex of Scrib, Discs-large (Dlg), and Lethal giant larvae (Lgl) [1].

**Figure 1:**
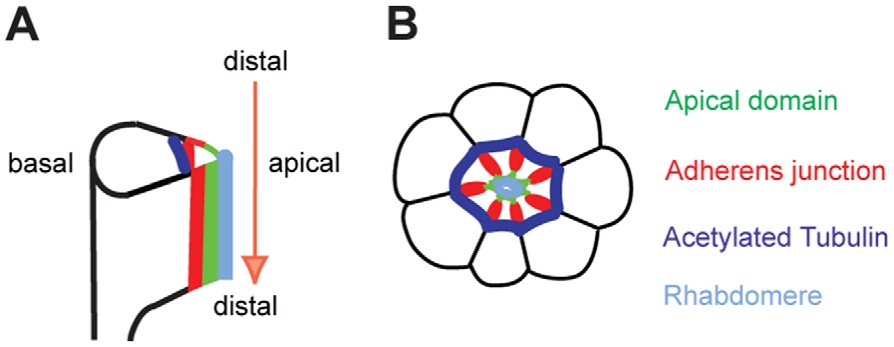
Morphogenesis of *Drosophila* pupal photoreceptors. (A) Side view of developing photoreceptors at 45% pd. The photoreceptors elongate from distal to proximal (arrow). (B) Cross-section of 45% pd pupal photoreceptors. Apical domain (green) localizes apical to adherens junction (red) in the center of a photoreceptor cluster. The E-cad localizes at adherens junction (red) which are more basal to the apical domain. The basolateral domains (black) are more basal to the adherens junction (red), and the acetylated-tubulin (blue) localizes at the outside from the adherens junctions (red). The rhabdomere (light blue) localizes at the apical to the apical domain (green).

Evolutionary conservation in the structure and function of polarity genes makes the *Drosophila* eye an excellent model for studying the genetic and molecular basis of retinal cell organization [2], [3]. Photoreceptor cells in *Drosophila* are formed in eye imaginal discs of third-instar larvae [4]. During pupal development, photoreceptor cells undergo the proximal to distal elongation (Fig 1A) while the apical membrane domain localizes at the center of the photoreceptor clusters surrounded by the adherens junction and the basolateral domains (Fig 1B) [5], [6]. Crb is required for extension of photoreceptors along the distal-proximal axis of the photoreceptor cell [2], [3]. The mammalian homolog of Crb, CRB1, is also localized to the inner segment of photoreceptors, the structure analogous to the rhabdomere stalk, between the outer segment and the adherens junction [3]. Furthermore, mutations in *CRB1* causes retinal diseases including retinitis pigmentosa 12 and Leber Congenital Amaurosis in humans [7], [8]. One of the important questions is how the apical Crb/CRB1 is regulated during the photoreceptor morphogenesis. Searching for new genes that interact with Crb will help in understanding the Crb-dependent eye development and degeneration. Here, we found that *spectraplakin* is one of the genes that affect Crb localization in the eye.

Microtubule cytoskeletons play essential roles in determining cell shape, cell polarity, and vesicle trafficking. As a consequence, microtubule reorganization during differentiation is essential for morphogenesis. Despite their importance in cell shape and polarity generation, the organization of microtubules in *Drosophila* photoreceptors remains relatively unexplored [9], [10], [11]. Therefore, we have recently examined the presence of stabilized microtubules in developing pupal photoreceptors and proposed its potential role in *Drosophila* photoreceptor development [12], [13]. We identified the presence of stable/acetylated microtubules in developing *Drosophila* pupal photoreceptors (Fig 1) [12], [13]. It was also found that Spastin, a microtubule-severing ATPase involved in constructing microtubule arrays, helps control the apical localization of Crb [12]. Furthermore, the microtubule-based motors, Kinesin-1 and Kinesin-2, were discovered to involve the Crb localization in developing photoreceptors [14], [15]. Therefore, Spectraplakin, an actin-microtubule cross-linker [16], might have a potential role in the regulation of stable-microtubules and apical Crb during the photoreceptors morphogenesis.

Spectraplakins are a family of actin-microtubule cross-linking proteins that have been highly conserved throughout the animal kingdom [16], [17]. All spectraplakins possess a series of plakin domains that are structurally similar to spectrin repeats. Many also have one or two amino-terminal calponin homology domains, a carboxy-terminal region containing a GAS2-like domain. The calponin homology domains, as two tandem copies, are able to bind tightly to actin filaments, whereas the GAS2 domain interacts with microtubules [18], [19]. Thus, spectraplakins are thought to be molecules with actin- and microtubule-binding sites on either end. This organization suggests that these molecules are able to act as actin-microtubule cross-linkers.

The Spectraplakin family is comprised of mammalian BPAG1/dystonin and ACF7/MACF1, *Drosophila* Short stop (Shot), and *Caenorhabditis elegans* Vab-10 [16], [18], [20], [21]. Spectraplakins have important roles in a broad spectrum of cellular contexts, ranging from highly dynamic roles in development or wound healing to structural functions in cell or tissue maintenance [17]. In this study, we characterized the role of Spectraplakin/Shot in photoreceptor development and examined the genetic interaction between the *spectraplakin/shot* and *crb*, a cell polarity gene which provides a positional cue for photoreceptor morphogenesis[2], [3].

## Results

### Genetic interaction between *crb* and *spectraplakin*/*shot* in *Drosophila* photoreceptors

**Figure 2:**
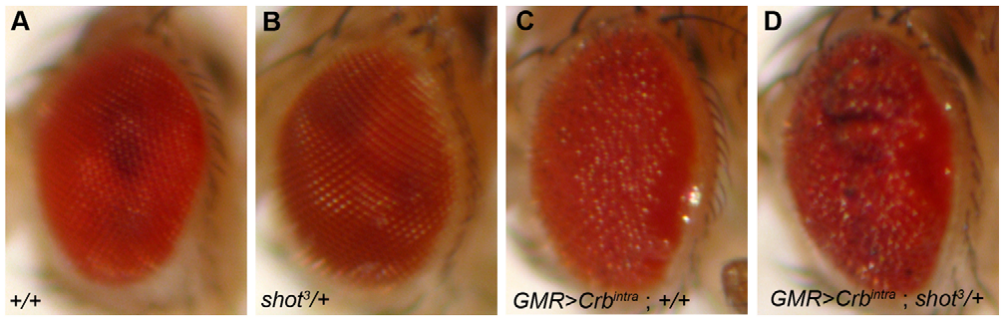
Genetic interactions between *crb* and *shot* in *Drosophila eye*. (A–B) Adult eye phenotypes of+/+(A), *shot^3^/+*(B), *GMR>Crb^intra^*;+/+(C), and *GMR>Crb^intra^*; *shot^3^/+*(D).

We overexpressed the conserved Crb intracellular domain (*Crb^intra^*) [22] using *GMR-Gal4* [23], which led to a roughening of the eye's external morphology (Fig 2C) [2]. Using this genetically sensitized condition, we performed a genetic screen to identify additional players that function with Crb to regulate photoreceptor morphogenesis. From a pilot screen, we found that the rough eye phenotype of *GMR>Crb^intra^* was dominantly enhanced by reducing the level of *shot* in the *shot/+*heterozygous background (Fig 2D), thus suggesting a strong genetic interaction between *crb* and *shot* in the *Drosophila* eye. The enhancement of the rough eye phenotype in the *shot/+*heterozygous background was very consistent with 100% penetrance (n>100). This genetic interaction data strongly suggests that Shot may provide an additional positional cue for Crb-dependent photoreceptor development.

### Localization of *Spectraplakin*/S*hot* in *Drosophila* pupal photoreceptors

**Figure 3:**
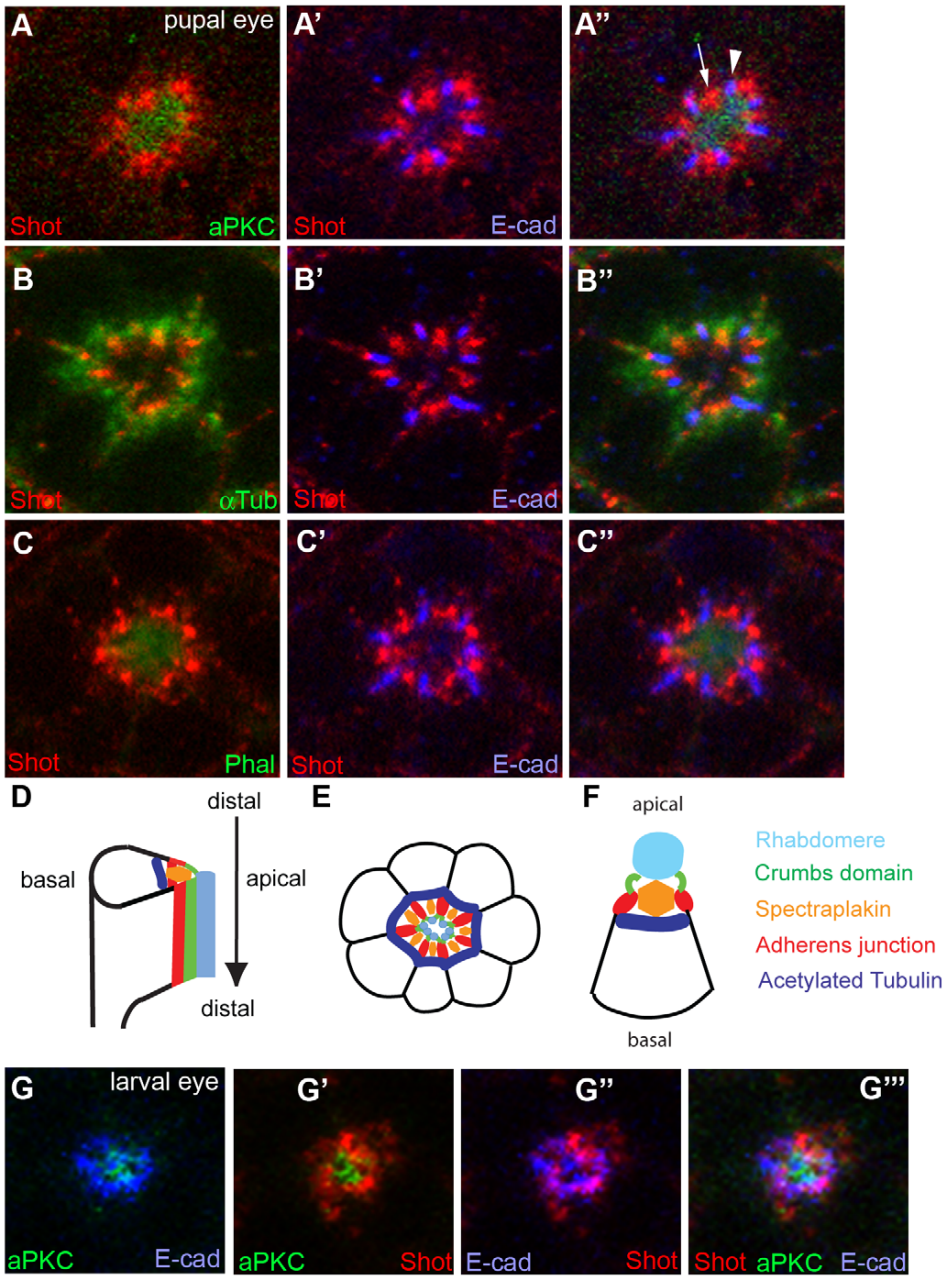
Localization of Shot in *Drosophila* photoreceptors. (A–F) Localization of Shot in pupal photoreceptors (45% pd). (A and B) Shot (A and B, red) localizes in between the E-cad (A' and B', blue), at the basal side of the aPKC (apical marker, A, green), and at the apical side of the microtubules (B, green). (C) Shot localizes at the basal side of phalloidin (Phal, rhabdomere marker, C and C''). (D–F) Schematic representation of mid-pupal photoreceptor and localization of Shot was labeled (orange). Shot (orange) localizes in between adherens junction (red), at the basal side of the apical Crumbs domain (green), at the apical side of the stable microtubule (blue), and at the basal side of the rhabdomere (light blue). (G) Localization of Shot in eye discs of third-instar larvae. Shot localizes at the basal side of the apical domain (aPKC, G'), and in between adherens junction (E-cad, G'').

After finding genetic interaction between *crb* and *shot*, it was then necessary to determine the localization of Shot in developing wild type photoreceptors. We utilized the anti-Shot monoclonal antibody, mAb Rod1 (Developmental Studies Hybridoma Bank, DSHB of Univ of Iowa) [24] to examine the localization of Shot in the mid-stage developing pupal eyes (45% pupal development stage, pd), which was examined by immunostaining and confocal microscopy. Previously, Shot was reported to localize at adherens junctions in embryonic and follicular epithelia cells [20], or with microtubules in oocytes [25]. We investigated where Shot localizes compared to other subcellular markers of aPKC (apical membrane domain) and E-cadherin (adherens junction) [26], [27], [28], and cytoskeleton markers of acetylated tubulin (stable microtubule) [12], [29] and phalloidin (F-actin, rhabdomere) [4] in mid-stage pupal photoreceptors. In *Drosophila* pupal photoreceptors, Shot is highly enriched in between the adherens junctions (E-cad), at the basal side of the Crb, and at the apical side of the microtubules (Fig 3A and 3B). This data strongly indicates that Shot is concentrated at the rhabdomere terminal web (RTW) [30], [31], [32], [33] in the developing photoreceptors. The RTW is considered to serve as a transition zone for the constant delivery of proteins needed for the maintenance and function of the rhabdomere [30], [31], [32], [33]. The “RTW” might be the place where the stable microtubules and fibrous actins (F-actins) meet (Fig 3D–3F), based on the localization of Shot, an actin-microtubule cross-linker. Therefore, Shot might have a potential role in the stable microtubules and rhabdomere, and thereby in the localizations of Crb and adherens junctions in photoreceptor cells (Fig 3D–3F). It is possible that the differential localization of Shot from the apical and adherens junction could be the cell-type and developmental-stage specific. The localization of Shot at the RTW in the pupal stage could be mainly due to the actin-microtubule junctions. Therefore, it is possible, the Shot localizes at a different location in the absence of the fibrous-actin of rhabdomere which is formed during the mid-stage pupal eyes [5], [6]. However, the differential localization pattern of Shot from aPKC and E-cad was also observed in the eye discs of third-instar larvae (Fig 3G). Therefore, the localization of Shot at the RTW in pupal eyes might be independent of the rhabdomere.

### Loss-of-function analysis of *spectraplakin*/s*hot* in *Drosophila* pupal photoreceptors

**Figure 4:**
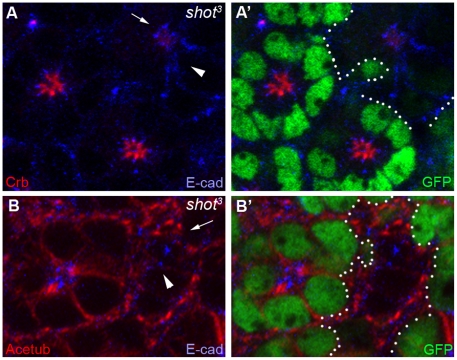
Shot is essential for photoreceptor morphogenesis in the mid-stage developing pupal eyes. (A and B) Mid-stage pupal eyes (45% pd) with *shot^3^* null mutant clones marked by the absence of the GFP and dotted lines (green, A' and B'). Crb (red, A, arrow) was almost absent, and E-cad (blue, A, arrowhead) was mislocalized from the apical center toward the surrounded distal areas (arrow, A). Stable microtubules (Acetub, red) were also mislocalized in the *shot^3^* mutants (arrow, B).

To examine whether Shot is required for photoreceptor morphogenesis in mid-stage pupal eye development, we generated mosaic eyes of a null mutation of *shot*, *shot^3^* [34], [35], using a genetic mosaic technique of FLP/FRT [36]. The allele *shot^3^* is genetically an amorphic/null allele that lacks Shot protein expression [20] and has been completely rescued by the *UAS-ShotA* transgene [19]. In the absence of Shot in *shot^3^* mutants, the apical Crb domain (Fig 4A, arrow) was dramatically reduced, and adherens junction (Fig 4A, arrowhead, E-cad, blue) was mislocalized from the apical center toward the basolateral areas (Fig 4). Furthermore, stable microtubules [12] were also disrupted (Fig 4B). These mutational analyses of the null allele of *shot* mutation strongly indicated that the Shot is indispensable in the photoreceptor morphogenesis, and is required for the correct positioning and/or targeting of apical domain, adherens junctions and stable microtubules during the photoreceptor development.

### Gain-of-function analysis of *spectraplakin*/s*hot* in *Drosophila* pupal photoreceptors

The loss-of-function analysis of the *shot* mutation (Fig 4) strongly suggests that *shot* is essential to maintain the apical Crb domain, adherens junctions, and stable microtubules during photoreceptor development. Therefore we hypothesize that Shot might have an active role for the positioning of Crb/E-cad/Acetub in photoreceptors. To test this hypothesis, a gain-of-function analysis of *shot* was conducted using an eye-specific Gal4 driver, *GMR-Gal4* [23], in order to increase *shot* expression in the developing photoreceptors.

**Figure 5:**
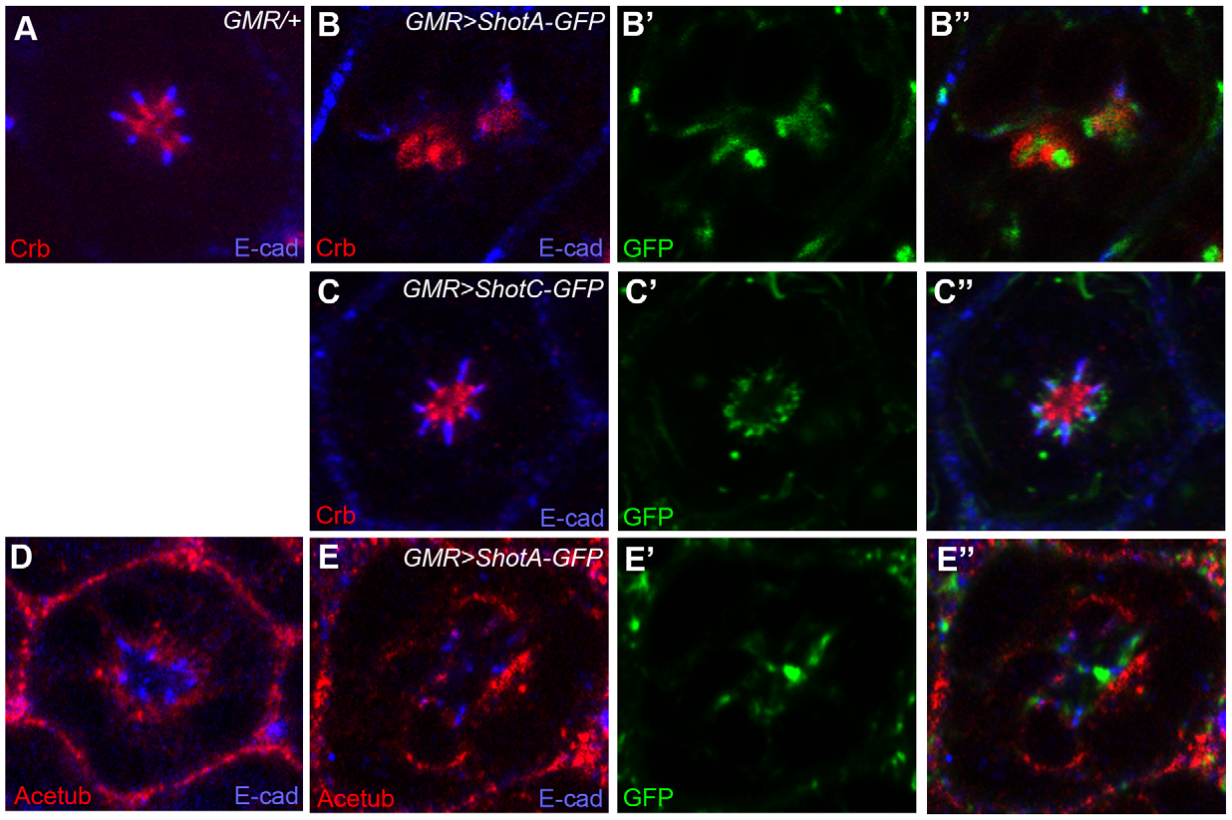
Overexpression of Shot causes the mislocalization of apical domain, adherens junction, and stable microtubules. Pupal eyes (45% pd) with Shot overexpression driven by *GMR-GAL4* at 22°C were examined by Crb (apical domain marker, red, A–C), E-cad (adherens junction marker, A–E) and acetylated-tubulin (Acetub, stable microtubules marker, red, D–E). (A, D) control, *GMR-GAL4/+*, (B, E) *GMR-GAL4/UAS-ShotA-GFP*, (C) *GMR-GAL4/UAS-ShotC-GFP*.

Several isoforms of Shot are ShotA, ShotB, and ShotC based on the alternative RNA splicing from the unique *shot* gene in *Drosophila* [16]. The Shot isoforms vary at their amino-terminus while having identical central and carboxy-terminal domains. The ShotC isoform differs from ShotA by lacking one calponin homology domain which is necessary for actin-binding ability. Therefore, ShotA has an actin-binding activity, but ShotC does not [18], [19]. The previously established *UAS-ShotA-GFP* and *UAS-ShotC-GFP* were used to test the effects of *Shot* overexpression in photoreceptor development. Overexpression of *ShotA* using *GMR-Gal4* in mid-stage pupal photoreceptors resulted in the dramatic mislocalized distribution of ShotA-GFP. In this situation, concurrent mislocalizations of Crb and E-cad (Fig 5B) occurred in the situation of ShotA-GFP overexpression. The Shot, Crb and E-cad were mingled together in this mislocalized situation (Fig 5B''). These results suggest that the ShotA-overexpression induce the complete cell polarity defects including the mislocalizations of the Crb and E-cad.

In the condition of ShotA-GFP overexpression, the most dramatic mislocalization happened in the localization of stable microtubules (Fig 5E). The stable microtubules were completely displaced from the apical areas to the basolateral areas in the ShotA-GFP overexpression situation (Fig 5E). Therefore, the primary target of Shot might be the stable microtubules. Then, the defected microtubules might further affect the Crb and E-cad, but other possibilities of direct targeting of Shot toward Crb and/or E-cad cannot be excluded.

When the ShotC-GFP, lacking the actin-binding activity, was expressed, the ShotC-GFP precisely localized at the “RTW” in the photoreceptors, and did not cause the mislocalization defects in Crb and E-cad (Fig 5C), although the expression level of ShotA-GFP and ShotC-GFP were similar based on the level of GFP tag (Fig 5B' and 5C'). These data suggest that the ShotA-induced mislocalizations of Crb/E-cad/Acetub require the active calponin homology domain for actin-binding activity. However, the targeting of Shot to the RTW might not require the calponin homology domain having the actin-binding activity, because the ShotC isoform localizes at the RTW without the actin-binding activity.

In conclusion, the role of Shot in Crb, E-cad and stable microtubules during photoreceptor development was identified, based on the loss-of-function (Fig 4) and gain-of-function (Fig 5) phenotypes of *shot* mutations.

### *crb* is required in Shot localization during *Drosophila* photoreceptor elongation

**Figure 6:**
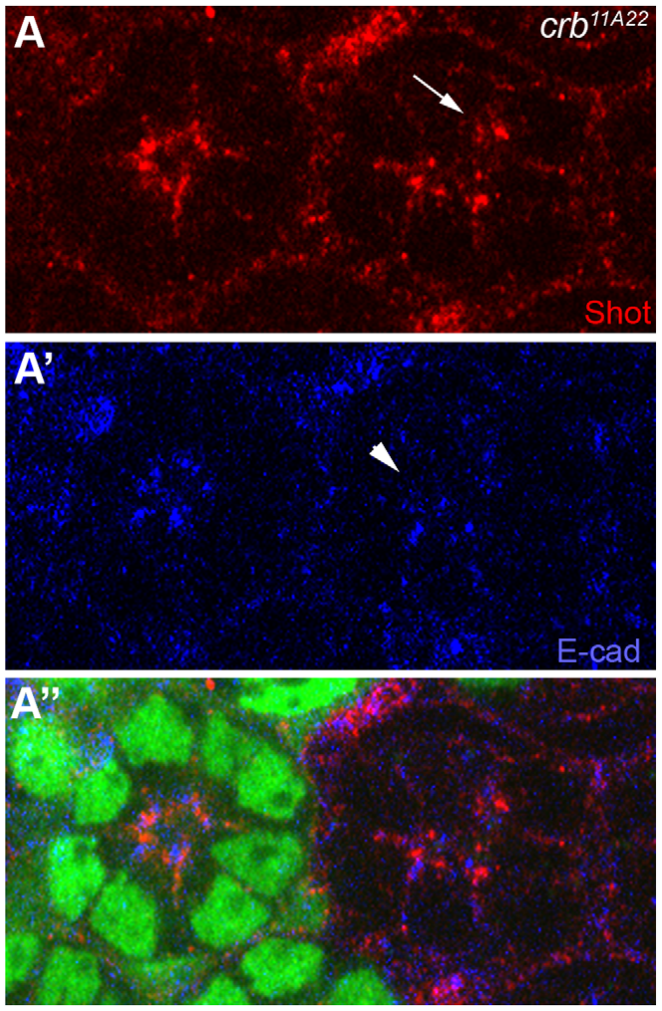
Crb is essential for Shot localization in the mid-stage developing pupal eyes. (A) Pupal eyes (45% pupal stage) with *crb^11A22^* null mutant clones marked by the absence of the GFP (green, A''). (A) Shot (red, A, arrow) and adherens junction (E-cad, blue, A', arrowhead) were mislocalized from apical to basal (A and A', arrow and arrowhead).

The genetic interaction between *shot* and *crb*, shows that the *shot/+* heterozygote dominantly enhanced *crb* gain-of-function phenotype (Fig 2). This genetic interaction data suggests that Shot may provide an additional positional cue for Crb-dependent photoreceptor development. Furthermore, *shot* mutation analysis shows Shot's role in the Crb's localization in photoreceptors (Fig 4 and 5). Therefore , it is necessary to analyze Crb's role in the Shot's localization in photoreceptors. We generated mosaic eyes of a null mutation of *crb*, *crb^11A22^* [37], using a genetic mosaic technique [36]. The allele *crb^11A22^* is genetically an amorphic/null allele and lacks Crb protein expression [2], [26], [37]. In the *crb* null mutants, the E-cad of adherens junction marker was mislocalized from apical to basal (Fig 6A', arrowhead) as previously reported [2], [3]. The Shot localization was also mislocalized in the *crb* mutants (Fig 6A, arrow), as much as the E-cad (Fig 6A', arrowhead). This data indicates that Crb is essential for Shot localization at the “RTW” in the photoreceptors.

## Discussion

We investigated where Shot localizes compared to apical membrane domain, adherens junction, stable microtubule, and rhabdomere (Fig 3), in mid-stage pupal photoreceptors. The localization results of Shot in pupal eyes strongly indicate that Shot localizes in between the adherens junctions, at the basal side of the apical domain, and at the apical side of the stable microtubules (Fig 3). The RTW, where the Shot localizes, may be the interface where the stable microtubules and F-actins of rhabdomere meet together. Since Shot has an actin-microtubule cross-linking activity, Shot might cross-link the two cytoskeletons of actin and microtubules at the RTW.

This genetic interaction data of *shot* and *crb* (Fig 2) strongly suggests that Shot may provide an additional cue for Crb in photoreceptor development because the rough-eye phenotype caused by Crb overexpression was further enhanced by reduced *shot* gene dosage (*shot/+*). The relationship between *crb* and *shot* might be one of the following possibilities; (i) *shot* acts at the upstream of *crb*, (ii) *shot* acts at the downstream of *crb*, or (iii) *shot* and *crb* control the parallel pathway in photoreceptor development. From comparative genetic analysis (Fig 4 and 6), Crb and Shot require each other reciprocally to localize at their target sites of rhabdomere stalk (apical domain) and RTW.

Our genetic analysis of the *shot* (Fig 4) mutation strongly indicates that Shot modulates the apical Crb membrane domain during rhabdomere elongation. The apical membrane modulation activity of *spectraplakin* was further confirmed by *spectraplakin* overexpression which caused a dramatic misplacement of the apical membrane domain (Fig 5). It is postulated that the spectraplakin might affect the actin (rhabdomere) and/or microtubules based on its dual binding capacity for actin/microtubule, and its activity as an actin/microtubule cross-linker [16]. Therefore, its role in photoreceptor morphogenesis might require its dual actin/microtubule binding, which will be supported by the absence of Crb-mislocalization activity of ShotC, which lacks the actin-binding domain. The Crb-mislocalization activity of ShotA might be based on its dual actin/microtubule binding ability.

One important point of the potential cross-talk between the *crb* and *shot* is their different spatial localizations in photoreceptors. How does one protein affect the other that is at a different localization in the cell? There might be at least several following possibilities; (i) a potential interaction when they co-localize during the trafficking before their final targeting, (ii) a potential interaction in previous developmental time, or (iii) a potential interaction at the interface where the two subcellular compartments meet. The “RTW” is the place where the microtubules and F-actins (rhabdomere) meet each other. Therefore, the Shot might have a potential role in the regulation of stable microtubules and rhabdomere, and thereby the localizations of Crb and adherens junctions might be affected in photoreceptor cells.

Shot has a microtubule organizing activity. Therefore, the expected result in the loss of *shot* is the defects of stable microtubules which were observed in the loss-of-function study of *shot* mutation (Fig 4B). Furthermore, the stable microtubules were mostly defected in the gain-of-function of ShotA-GFP overexpression (Fig 5E). Therefore, the primary target of Shot seems to be the stable microtubules. Then, the defected microtubules may further affect the Crb and E-cad through the microtubule-based trafficking [14], [15] and/or other microtubule-based cell polarity [38]. But the other possibilities of direct targeting of Shot toward Crb/E-cad or actin-based cell polarity cannot be excluded. ShotA-GFP overexpression results in ectopic localization of Acetub around the cells (Fig 5E) which could be caused by the direct binding of ShotA-GFP to the Acetub. However, another possibility of the indirect mislocalization of Acetub caused by the mislocalized “RTW” by the ShotA-GFP cannot be excluded.

Another potential possibility of Spectraplakin's function in the regulation of apical membrane domain might be through microtubule plus-end-tracking proteins (+TIPS). The +TIPS belong to the class of microtubule-associated proteins, and link microtubule ends with apical actin cytoskeleton [39], [40]. In *Drosophila* muscle-tendon junctions, Shot regulates microtubule cytoskeleton by forming a complex with the EB1 and APC of +TIPS [41], [42]. Therefore, there is a potential possibility of Shot and +TIPS interaction in apical domain control during photoreceptor morphogenesis.

We have shown that Spectraplakin/Shot is required for correct localization of Crb, adherens junctions, and stable microtubules in the photoreceptors and disruption of Shot function affects photoreceptor morphogenesis. Our data strongly suggests that Spectraplakin/Shot plays important functions in the modulation of cell membrane domains including the apical Crb domains of photoreceptors during pupal eye development. Evolutionary conservation in the structure and function of eye morphogenesis genes makes the *Drosophila* eye an excellent model for studying the genetic and molecular basis of retinal cell organization. Future work will help to uncover other genes that might affect the Crb positioning during the extensive morphological growth phase of the *Drosophila* pupal eye. Given the high degree of evolutionary conservation of Crb and Spectraplakin genes from *Drosophila* to higher mammals including humans, similar cooperative mechanism between Crb and Spectraplkain could have a role in the development and degeneration of human photoreceptor.

## Materials and Methods

### Genetics

All *Drosophila* strains were grown and maintained at room temperature. Mitotic recombination was induced by using the FLP/FRT method for clonal analysis [36]. *shot^3^* is a null allele lacking detectable Shot protein [34], and has been completely rescued by the *UAS-ShotA* transgene [19]. *shot^3^* mutant clones were produced by eye-specific expression of ey-Flp [43] or *GMR-flp* [44] in *y w ey-Flp* or *GMR-flp/+; FRT42D shot^3^ / FRT42D Ubi-GFP*, or *y w ey-Flp/+; FRTG13 shot^3^ / FRTG13 Ubi-GFP*. *crb^11A22^* is a null allele of *crb* [36]. *crb^11A22^* mutant clones were produced in *y w ey-Flp/+; FRT82D crb^11A22^ / FRT82D Ubi-GFP*. Overexpression of Shot was induced by crossing *UAS-ShotA-GFP* or *UAS-ShotC-GFP* [19] with *GMR-GAL4* [23] at room temperature (22°C). *shot^3^*, *UAS-ShotA-GFP*, *UAS-ShotC-GFP*, and *GMR-Gal4* were obtained from the Bloomington Stock Center at Indiana University.

### Immunohistochemistry

Fluorescent immunostaining and confocal analysis of pupal eyes were carried out as reported [12], [13], [14], [26], [27], [28], [29]. Dissected pupal eyes were fixed for 20 min at room temperature in 4% paraformaldehyde. A 15 minute acetone treatment was performed after fixation for rat anti-Crb staining [45]. The fixed pupal eyes were permeabilized by incubation for 10 min at room temperature with 0.5% NP-40 in 50 mM Tris-Cl (pH 6.8), 150 mM NaCl, and 5 mg/ml BSA. The tissues were then incubated overnight at 4°C to primary antibodies in 0.5% NP-40 in 50 mM Tris-Cl (pH 6.8), 150 mM NaCl, and 1 mg/ml BSA. The following primary antibodies were used: mouse anti-acetylated tubulin (Sigma), 1∶1000; rabbit anti-α-tubulin (Abcam), 1∶200, rat anti-E-cadherin (Dcad2, DSHB) [46], 1∶20; mouse anti-Shot (Rod1, DSHB) [24], 1∶20; rat anti-Crb [45], 1∶400; sheep anti-GFP (Biogenesis), 1∶100; rabbit anti-aPKCζ (Santa Cruz), 1∶500. Immune complexes were detected by incubation for 4 hrs at 4°C with donkey Cy3-, Cy5-, or FITC-conjugated secondary antibodies (Jackson ImmunoResearch). F-actin was stained by FITC-phalloidin (Sigma) or TRITC-phalloidin (Sigma) at a concentration of 1 µM. The stained tissues were mounted in Vectashield (Vector Laboratories). Fluorescent images were acquired on an Olympus FV 1000 confocal microscope (Olympus) equipped with a 60x oil-immersion objective lens (Plan-Aprochromat, NA 1.42, WD 0.15 mm). The image analysis and quantification were analyzed using ImageJ (NIH). The size, contrast and brightness of the resulting images were adjusted with Photoshop CS (Adobe Systems).
